# The role of endogenous hormones in regulating grain number in oat panicles under drought stress across cultivars

**DOI:** 10.3389/fpls.2026.1808726

**Published:** 2026-05-08

**Authors:** Jiahao Li, Xingyu Wang, Jinghui Liu, Junzhen Mi, Xiquan Wang, Baoping Zhao

**Affiliations:** College of Agronomy/National Outstanding Talents In Agricultural Research And Its Innovation Team, Inner Mongolia Agricultural University, Hohhot, China

**Keywords:** *Avena sativa* L., cytokinin, grain number, grain yield, indole-3-acetic acid

## Abstract

**Introduction:**

Drought stress poses a major constraint on oat (*Avena sativa* L.) yield formation. Endogenous phytohormones play a crucial role in the adaptive mechanisms of oat. However, investigating the distinct parts of the oat panicle in relation to endogenous hormones remains a research gap for understanding the physiological mechanisms underlying yield formation.

**Methods:**

A two-year field experiment was conducted using ten oat cultivars to investigate the interaction between yield traits in the upper and lower parts of the oat panicle under drought stress.

**Results:**

The ten cultivars were classified into two groups based on their drought resistance index (DI): drought-tolerant cultivars (DI ≥ 0.670) and drought-sensitive cultivars (DI ≤ 0.574). Regardless of whether cultivars were drought-tolerant or drought-sensitive, grain number per panicle served as the principal determinant of yield (*R*^2^ = 0.182–0.368). Yield variation among oat cultivars was primarily attributed to the difference in grain number on the lower panicles, under both drought and well-watered conditions (*R*^2^ = 0.879–0.923). Under drought stress, cytokinin (CTK) content significantly decreased in drought-tolerant cultivars (*p* < 0.001), while a reduction was observed only in the upper panicles of the drought-sensitive cultivars (*p* < 0.05). Under drought stress, the indole-3-acetic acid (IAA) content in the lower panicles increased in drought-tolerant cultivars (*p* < 0.001), while it significantly decreased in drought-sensitive cultivars (*p* < 0.001).

**Discussion:**

These results demonstrate that IAA and CTK in drought-tolerant cultivars play a pivotal role in modulating basal grain development under water deficit conditions.

## 1 Introduction

Drought stress represents one of the most formidable constraints to global agricultural productivity, a challenge exacerbated by increasing climate variability (Ohlert et al., 2025). Its impact is particularly acute in cereal production systems, which form the cornerstone of world food security (Cho et al., 2022). Oat (*Avena sativa* L.), while renowned for its nutritional profile and adaptability to cooler climates and marginal soils, exhibits significant susceptibility to water deficit during critical reproductive stages (Nan et al., 2023; McCabe and Burke, 2021). Drought stress affects endogenous hormone contents in oat, leading to a reduction in grain number per panicle and consequently decreasing yield (Ferreira et al., 2023; Warzecha et al., 2023; Neupane et al., 2025). Understanding the mechanisms underpinning drought tolerance in oat is, therefore, not merely an academic pursuit but a pressing necessity for enhancing crop resilience and ensuring sustainable grain production under increasingly arid conditions.

The reproductive architecture of the oat plant, particularly the panicle, is not a homogeneous organ but a structure characterized by significant topological and physiological gradients (Berro et al., 2023; Steiner et al., 2017). It is well-established that grain development, filling, and final weight often vary systematically between the upper (apical) and lower (basal) portions of the panicle across cereal crops (Haverroth et al., 2021). This spatial disparity is often associated with differences in floral development timing, vascular connectivity, and, crucially, the localized hormonal microenvironment (Shanmugaraj et al., 2023). The concept of hormonal gradients dictating developmental fates is a fundamental principle in plant physiology, and its application to the cereal panicle provides a powerful framework for understanding yield determination (Zhang et al., 2024b and Zhang et al., 2024c). Previous research in other cereals has suggested that the resilience of specific panicle sections under stress may be linked to distinct hormonal buffering capacities (Chen et al., 2023; Rashid et al., 2024). However, in the context of oat, a critical knowledge gap persists. While bulk-tissue analyses have described general hormonal shifts under drought, they inevitably obscure the critical local dynamics occurring within different panicle sections (Li et al., 2025). How drought-tolerant oat cultivars differentially modulate the hormonal milieu in the upper versus lower panicles, compared to their drought-sensitive counterparts, remains largely unexplored. This gap is particularly relevant given that yield stability under drought stress is likely determined not by the plant’s overall hormone status, but by its ability to maintain conducive hormonal conditions in key reproductive structures.

Oat confronted with drought stress enact a suite of sophisticated physiological and biochemical adjustments. These include osmotic adjustment through the accumulation of compatible solutes, stomatal regulation to minimize water loss, and the reconfiguration of metabolic pathways to prioritize survival processes (Girija et al., 2025; Fang et al., 2024; Fei et al., 2025). Central to the coordination of these complex responses is the dynamic reprogramming of endogenous phytohormones (Li et al., 2025). These chemical messengers-including abscisic acid (ABA), cytokinin (CTK), indole-3-acetic acid (IAA), and gibberellins (GA) function as an intricate signaling network that translates the external stress stimulus into defined developmental and physiological outcomes (Ma et al., 2025; Liang et al., 2025; Peleg et al., 2011). ABA is widely recognized as the primary stress hormone, rapidly accumulating under water deficit to trigger stomatal closure and induce a state of physiological conservatism (Hsu et al., 2021; Liu et al., 2025). However, the plant’s overall response is not governed by ABA alone. A nuanced antagonistic and synergistic interplay, or “crosstalk,” among various hormones ultimately determines the balance between stress defense and growth maintenance (Waadt et al., 2022). For instance, while ABA promotes stomatal closure, CTKs are known to antagonize this process, and the IAA/CTK balance is critical for regulating meristem activity and organ development (Hussain et al., 2021).

This study is predicated on the hypothesis that the differential drought tolerance among oat cultivars is intrinsically linked to cultivar-specific, spatially-explicit hormonal remodeling within the panicle. We postulate that drought-tolerant cultivars employ a superior strategy by maintaining or enhancing a favorable IAA and CTK environment in the critical lower panicles, thereby preserving fertile floret development and grain sink strength under water deficit, whereas sensitive cultivars fail to do so. To test this hypothesis, we conducted a comprehensive two-year field study employing a diverse panel of ten oat cultivars, which were systematically classified into drought-tolerant and drought-sensitive groups based on a rigorous Drought Resistance Index (DI). The objectives of this investigation were threefold: 1) To identify the principal yield components and their spatial localization within the panicle that govern yield variation under drought stress. 2) To delineate the contrasting patterns of ABA, IAA, CTK and GA_3_ redistribution in the upper versus lower panicles in response to water deficit, with respect to cultivar tolerance. 3) To elucidate the pivotal role of these spatially-defined hormonal changes in modulating basal grain development and final yield determination. Through this integrated physiological approach, we aim to advance the understanding of drought tolerance mechanisms in oat by linking spatial hormone dynamics with yield architecture. The findings are expected to provide a physiological basis for future breeding strategies aimed at developing oat varieties with enhanced yield stability in drought-prone environments.

## 2 Materials and methods

### 2.1 Plant growth and treatments

**Figure 1:**
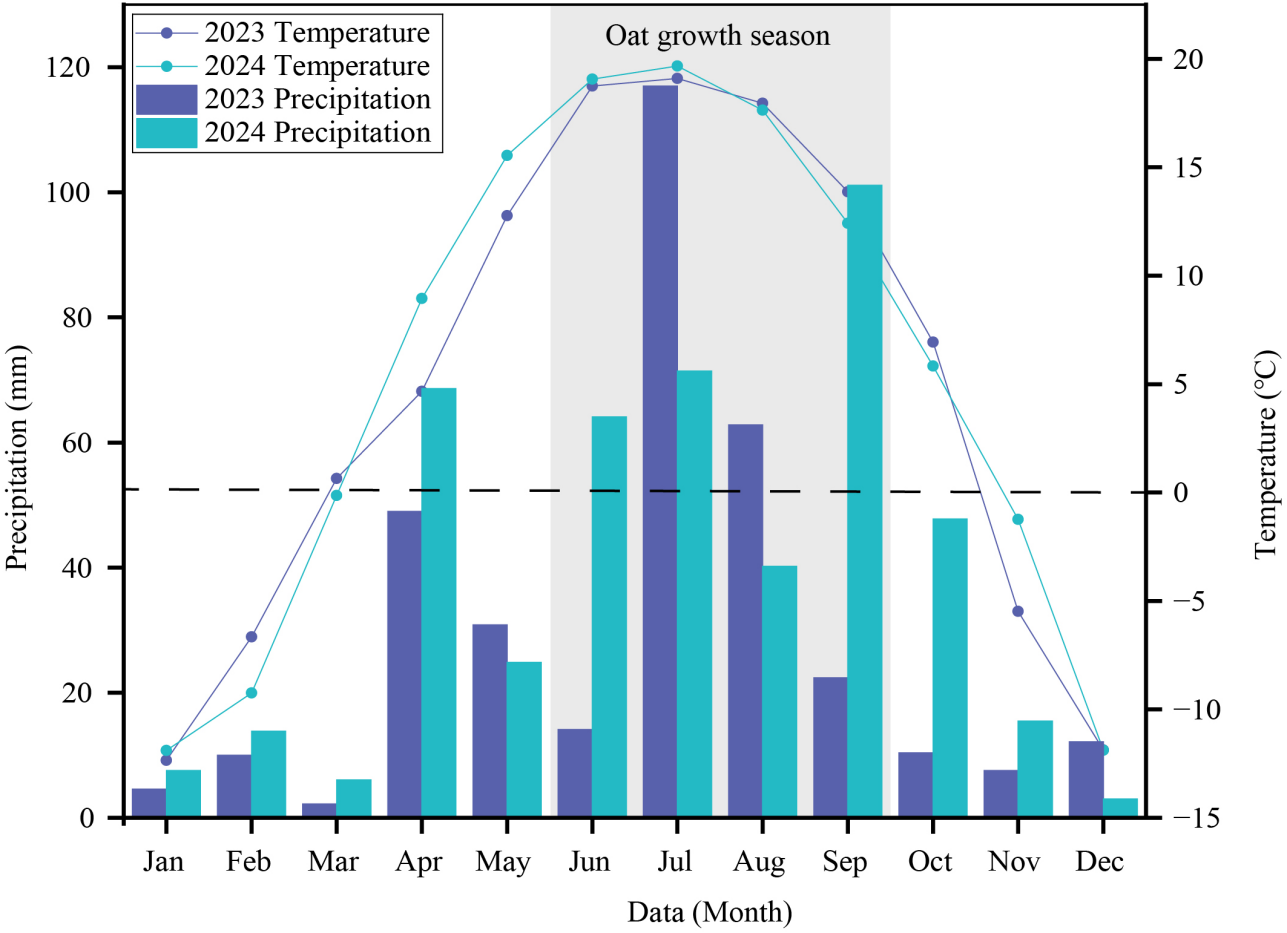
The mean monthly temperature and precipitation in 2023-2024. The dotted line represents that the mean monthly temperature is 0°C.

The experiment was carried out from 2023 to 2024 in the experimental field of the Oat Science and Technology Backyard in Wuchuan, Inner Mongolia. It is located in Shangtuhaixiang, Wuchuan County, Hohhot City (41°8′N, 111°21′E, 1627 m above sea level). It is a typical semi-arid agricultural area with an average annual precipitation of 363 mm, an average annual temperature of 4.2°C, and a frost-free period of about 120 days. The surface water resources in the area are scarce. The monthly average temperature and precipitation during the growth period of oats from 2023 to 2024 are shown in **Figure 1**, and the basic properties of the soil in the experimental field are shown in **Table 1**. We selected major oat cultivars released from the 1970s to the 2010s, which exhibit significant variation in grain number per panicle. The trial yield and grain number per panicle of the ten oat cultivars are presented in **Table 2**.

**Table 1:** Basic soil characteristics of experimental site in 2023-2024.

Year	Total N (g·kg^-1^)	Total P (g·kg^-1^)	Total K (g·kg^-1^)	Alkali Hydrolysable N (mg·kg^-1^)	Available P (mg·kg^-1^)	Exchangeable K (mg·kg^-1^)	SOC (g·kg^-1^)	pH
2023	1.12	0.79	17.98	101.06	16.57	207.29	10.43	8.32
2024	1.34	0.58	17.44	78.73	16.89	143.40	9.24	7.03

**Table 2:** Key characteristics of 10 oat cultivars.

No.	Cultivar	Yield (Mg·ha^-1^)	Growth period (days)	Breeding year
1	Dingyou 1	1.89	85-90	1970s
2	Bayou 1	2.25	85-95	1980s
3	Pin 5	1.96	85-90	1990s
4	Bayou 3	2.82	95-100	1990s
5	Bayou 9	2.91	80-85	2000s
6	Jinyan 14	2.47	90	2000s
7	Jinyan 17	2.29	95	2000s
8	Bayou 18	3.05	95-100	2010s
9	Zhangyou 9	2.76	90-100	2010s
10	Jinyan 20	2.80	90	2010s

The experiment was arranged in a split-plot design with three replications. The main-plot factor consisted of two water regimes: drought stress and well-watered control. Under the drought stress treatment, irrigation was applied only once prior to seedling emergence, whereas the well-watered control received irrigation at three growth stages: pre-emergence, jointing, and booting. The sub-plot factor comprised ten oat cultivars. In the well-watered treatment, irrigation was applied before seed emergence and at the jointing stage. In the drought stress treatment, irrigation was only carried out before the seedlings emerged. Each cultivar had three replicates. Each plot was designed to be 3m × 4m with a row spacing of 25 cm, and a total of 60 plots were set up. Micro-sprinkler irrigation was used. In the drought stress treatment, no irrigation was applied after sowing, relying entirely on natural precipitation. In the well-watered treatment, irrigation was carried out before sowing emerged and during the jointing stage, with the amount of irrigation being 60 mm. A small field weather station was used to record the air temperature and precipitation (**Figure 1**). The mean growing season accumulated temperature for oat was 2114°C, with a mean monthly precipitation of 61.6 mm. The compound fertilizer with a ratio of N: P: K = 18: 18: 18 was applied at a rate of 150 kg·ha^-1^, and for other management methods, conventional field management practices were adopted.

### 2.2 Grain yield and its components of oat

At physiological maturity, oat plants were collected to assess key yield components. From each plot, a 1 m² area was harvested to determine grain yield after threshing. Additionally, ten main stem panicles per cultivar per replicate were randomly selected for panicle length measurement. Another twenty oats per plot were sampled and divided into upper and lower parts to quantify the number of fertile and sterile florets, seed-setting rate, grains per panicle, and thousand-grain weight. These plant samples were then placed in paper bags, withered at 105°C for 20 minutes, and dried at 80°C to a constant weight.

The drought resistance index (DI) was calculated based on the yield performance under drought stress and well-watered conditions over two years. The calculation formulas are as follows:

(1) $$\text{DC}=\frac{{\text{Y}}_{\text{drought}}}{{\text{Y}}_{\text{irrigation}}}$$

(2) $${\bar{\text{Y}}}_{\text{drought}}=\sum_{\text{i}=1}^{\text{n}}{\text{Y}}_{\text{drought}}/\text{n}$$

(3) $$\text{DI}=\frac{\text{DC}\times {\text{Y}}_{\text{drought}}}{{\bar{\text{Y}}}_{\text{drought}}}$$

where:

DC is the drought coefficient of a single cultivar; $Y_{\text{drought}}$ is the yield of the genotype under drought stress; $Y_{\text{irrigation}}$ is the yield of the same cultivar under well-watered conditions; ${\bar{Y}}_{\text{drought}}$ is the mean yield of all cultivars under drought stress; n is the total number of cultivars; $Y_{\text{drought}}$ is the yield of one cultivar under drought stress; DI is the drought resistance index.

We calculated DI (Drought resistance index) according to Equations 1–3.

### 2.3 ABA, CTK, IAA, GA_3_ content

At the booting stage, a 1m² area was selected per plot, and 20 panicles were randomly sampled with four repetitions. The panicles were separated into top and bottom parts and stored in a -80°C ultra-low temperature freezer. The endogenous levels of ABA, CTK, IAA, and GA_3_ were extracted using 80% (v/v) methanol and quantified using enzyme-linked immunosorbent assay (ELISA) following the established methods in previous studies (Yang et al., 2001). The ELISA kits utilized in this research were provided by the Phytohormones Research Institute at China Agricultural University.

### 2.4 Statistical analysis

**Figure 2:**
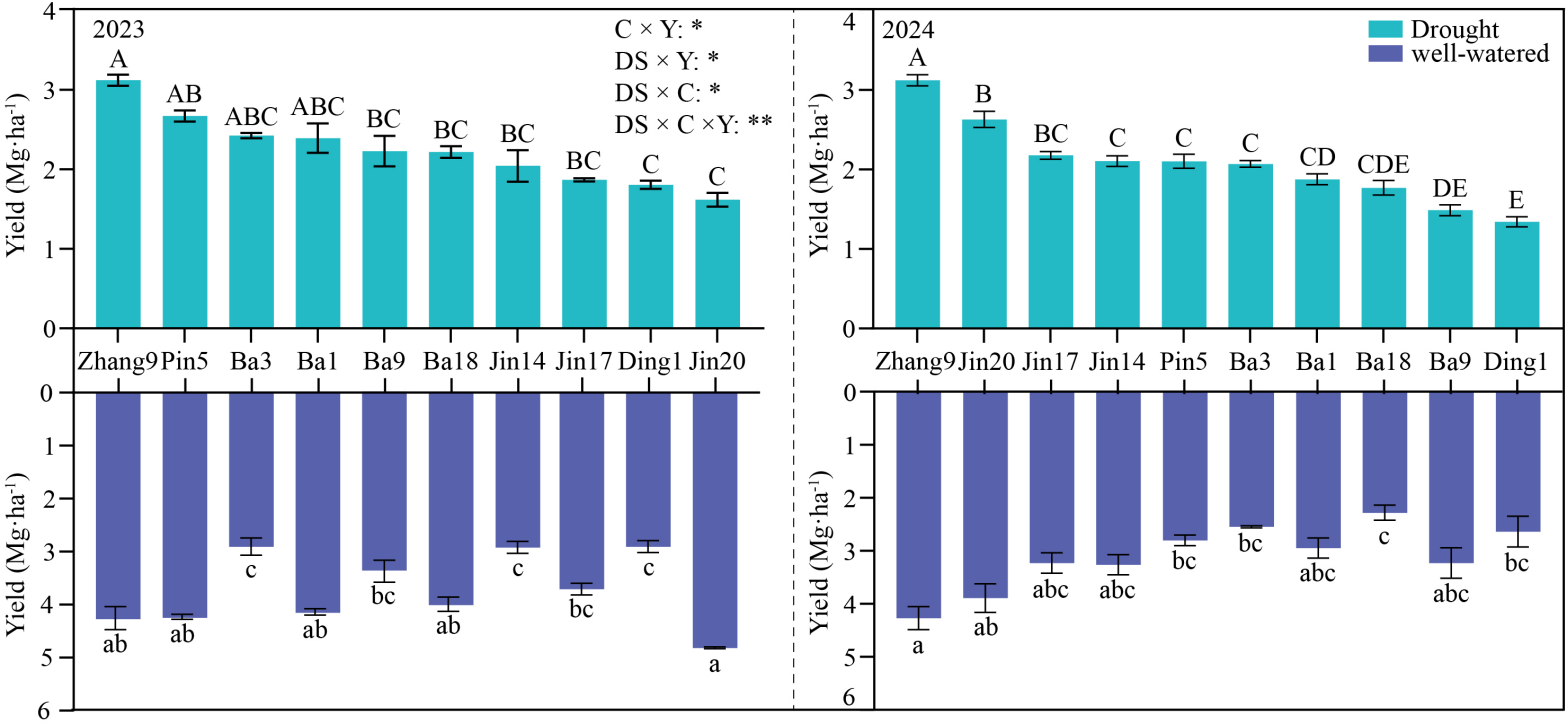
Grain yield of oat cultivars in 2023-2024. Error bars indicate standard error of the mean (n = 3). C × Y, DS × Y, DS × C and DS × C × Y are the F values of the variance analysis for cultivar, year and drought stress and their interaction effect, respectively. The symbol of * and ** indicate significant effect at 0.05 and 0.01 level, respectively. Capital letters are significantly different under drought treatment, and lowercase letters are significantly different under irrigation treatment (*p* < 0.05, Duncan’s test).

Each experimental unit was defined as a plot corresponding to a specific combination of cultivar, treatment, and replicate within each year. Measurements taken from multiple plants within the same plot were averaged to obtain a single value per experimental unit before statistical analysis. Year was treated as an independent factor, and the data from two years were analyzed separately or using a combined analysis across years, as specified in the figure legends. The Least Significant Difference (LSD) test for treatment comparisons, were performed using SPSS 27.0 (IBM, New York, NY, USA). The drought resistance coefficient data of the ten oat cultivars were subjected to hierarchical cluster analysis using SPSS 27.0 software. The clustering was performed using average linkage as the clustering method and Euclidean distance as the distance metric. The cluster centers were determined based on the total distance criterion. When the Euclidean distance threshold was set to 25, the cultivars were grouped into two distinct clusters (**Figure 2**). Linear regression models were conducted in GraphPad Prism version 9.0.0 (GraphPad Software, San Diego, CA, United States) to assess the relationships of yield and yield components, as well as the influence of grain number in upper and lower panicle on the total grain number per panicle. The differences in endogenous hormone concentrations among panicle parts of oat were analyzed by one-way ANOVA in combination with multiple comparisons Fisher’s Least Significant Difference (LSD) test at a significance level of *p* < 0.05. Box plots were created using GraphPad Prism 9.0 to visualize the distribution of the hormone data. Redundancy analysis (RDA) was conducted to assess the effects of endogenous hormone concentrations in upper and lower oat panicle on the grain number per panicle part and yield using the vegan package in R version 4.3.2 (R Foundation for Statistical Computing, Vienna, Austria). Random Forest analysis was built using the randomForest package in R (version 4.3.2). The grain number of the lower panicle was set as the response variable. Predictor variables comprised the concentrations of endogenous hormones measured in the upper, and lower parts of the panicle. Model performance and variable importance were assessed using the A3 package. The number of trees (ntree) was set to 1000, and the number of variables tried at each split (mtry) was tuned for optimal performance. The importance of each hormonal predictor was evaluated based on the %IncMSE (Percent Increase in Mean Squared Error, a measure of prediction accuracy loss when the variable is permuted).

## 3 Results

### 3.1 Oat grain yield

Compared with the well-watered control, drought stress significantly reduced grain yield in all tested oat cultivars, but the magnitude of reduction varied substantially by year and cultivar. In 2023, Jinyan 20 exhibited the largest yield reduction under drought stress (64.0%), whereas Zhangyou 9 showed the smallest reduction (4.1%). In 2024, Bayou 9 had the largest yield reduction (54.0%), while Zhangyou 9 again showed the smallest reduction (18.8%). These results indicate that Zhangyou 9 maintained relatively stable yield performance under drought across both years. Two-way ANOVA revealed significant interactions between treatment and cultivar, treatment and year, and cultivar and year (*p* < 0.05 for all). Moreover, the three-way interaction of treatment × cultivar × year was highly significant (**Figure 2**, *p* < 0.01), suggesting that the combined effects of drought, genotype, and growing season are complex and non-additive.

### 3.2 Oat drought resistance index

**Figure 3:**
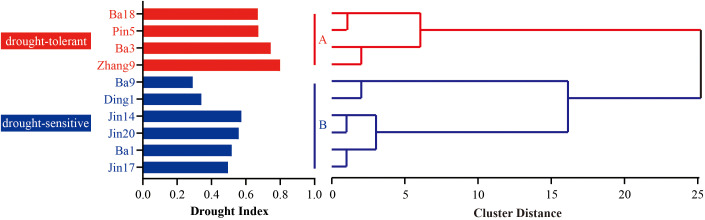
Cluster analysis results based on drought index (DI) in 2023-2024. The drought resistance ability of oats was divided into two categories, with the red portion denoted representing drought-tolerant cultivars, and the blue portion represents drought-sensitive group.

The drought resistance index (DI) of different oat cultivars over two years is presented in **Figure 3**. Based on a hierarchical clustering analysis with the Euclidean distance set at 25, the tested cultivars were clearly divided into two distinct categories. The first category comprised four drought-tolerant cultivars, namely Zhangyou 9, Pin 5, Bayou 3, and Bayou 18, with DI values ranging from 0.670 to 0.852. Among these, Zhangyou 9 exhibited the highest DI (0.852), indicating superior drought tolerance. The second category consisted of six drought-sensitive cultivars: Bayou 9, Dingyou 1, Jin 14, Jin 20, Ba 1, and Jin 17, with DI values ranging from 0.332 to 0.574. Compared with the drought-tolerant group, the sensitive cultivars showed DI values at least 0.096 lower. Notably, the DI threshold for differentiating the two groups was approximately 0.62, as the highest DI among sensitive cultivars was 0.574 (Jin 17) and the lowest among tolerant cultivars was 0.670 (Bayou 18).

### 3.3 Oat grain number in upper and lower panicle

**Figure 4:**
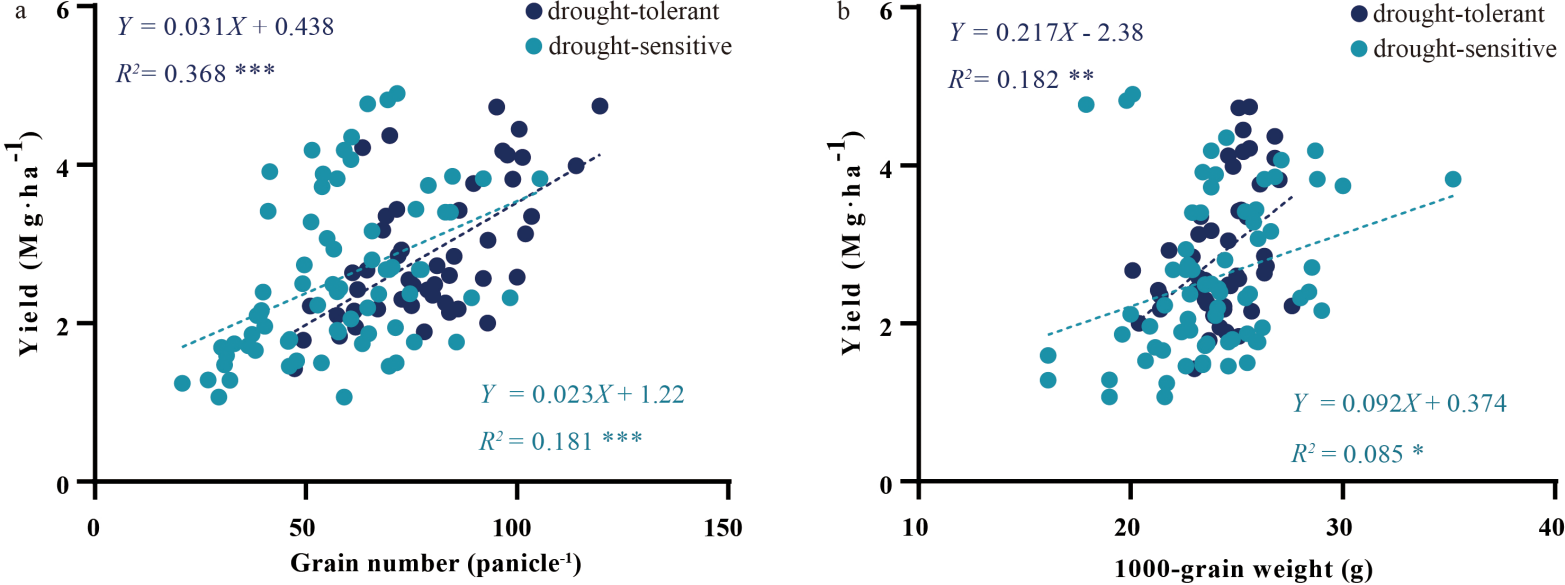
Linear relationships between yield components and grain yield in drought-tolerant and drought-sensitive oat cultivars. **(a)** grain number per panicle vs. grain yield in drought-tolerant and drought-sensitive cultivars; **(b)** 1000-grain weight vs. grain yield in drought-tolerant and drought-sensitive cultivars. Significance levels: *p < 0.05, **p < 0.01, ***p < 0.001.

Linear regression analysis revealed distinct yield-determining patterns between drought-tolerant and drought-sensitive oat cultivars. In drought-tolerant cultivars, grain yield was more closely associated with grain number per panicle (*R²* = 0.368 ***) than with grain weight (*R²* = 0.181 ***). The same trend was observed in the drought-sensitive cultivars, grain yield was more closely associated with grain number per panicle (*R²* = 0.182 **) than with grain weight (*R²* = 0.085 *). This indicates that grain number per panicle had a stronger influence on yield than grain weight across all cultivars (**Figure 4**).

**Figure 5:**
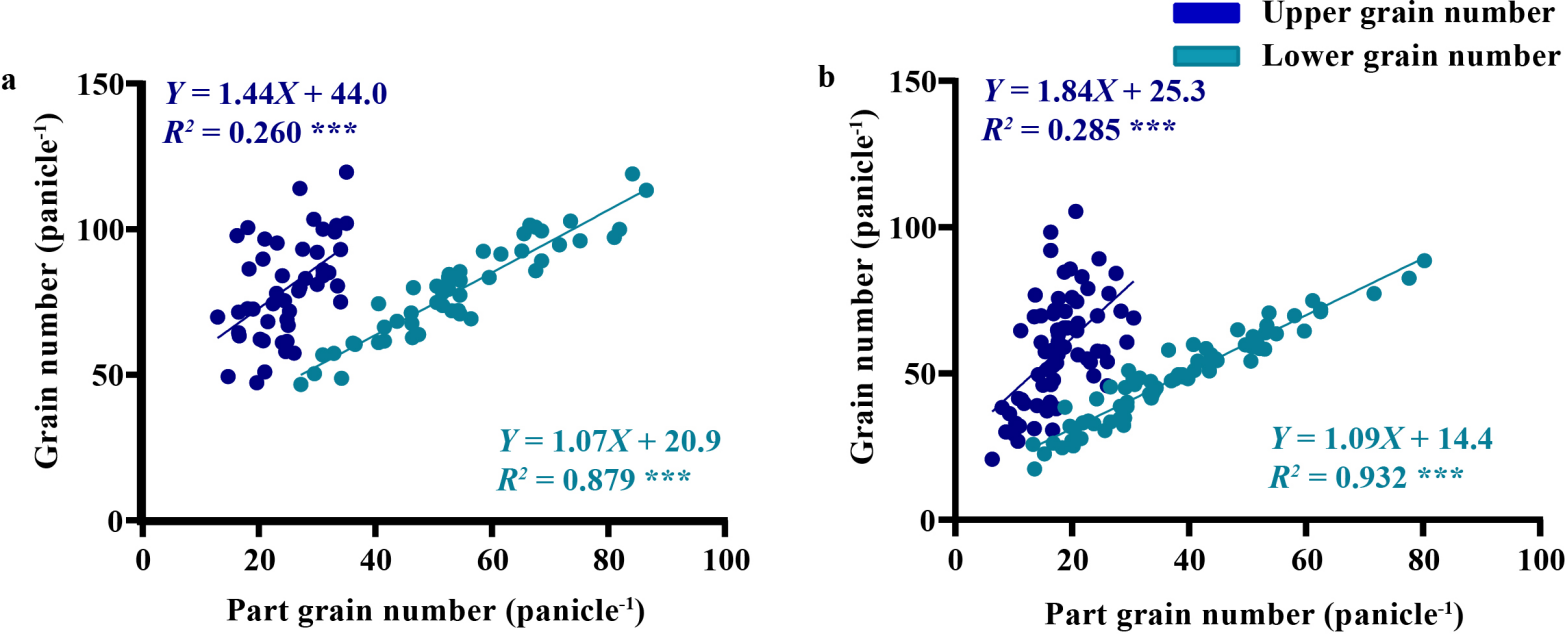
Linear relationships between grain numbers in different panicle positions and whole-plant grain number under drought stress and normal irrigation in high drought-tolerant vs. drought-sensitive oat cultivars. **(a)** linear relationship between grain numbers in different panicle positions and whole-plant grain number in drought-tolerant oat cultivar; **(b)** linear relationship between grain numbers in different panicle positions and whole-plant grain numbers in drought-sensitive oat cultivars. Significance levels: *p < 0.05, **p < 0.01, ***p < 0.001.

Regression models identified lower panicle grain number as the key yield predictor across all cultivars (*R²* = 0.879-0.923), with enhanced explanatory power under drought. This highlights its role as a conserved adaptive response to drought stress (**Figure 5**). This phenomenon may be attributed to intensified assimilate competition within lower panicles under drought stress.

### 3.4 ABA, CTK, IAA and GA_3_ content

**Figure 6:**
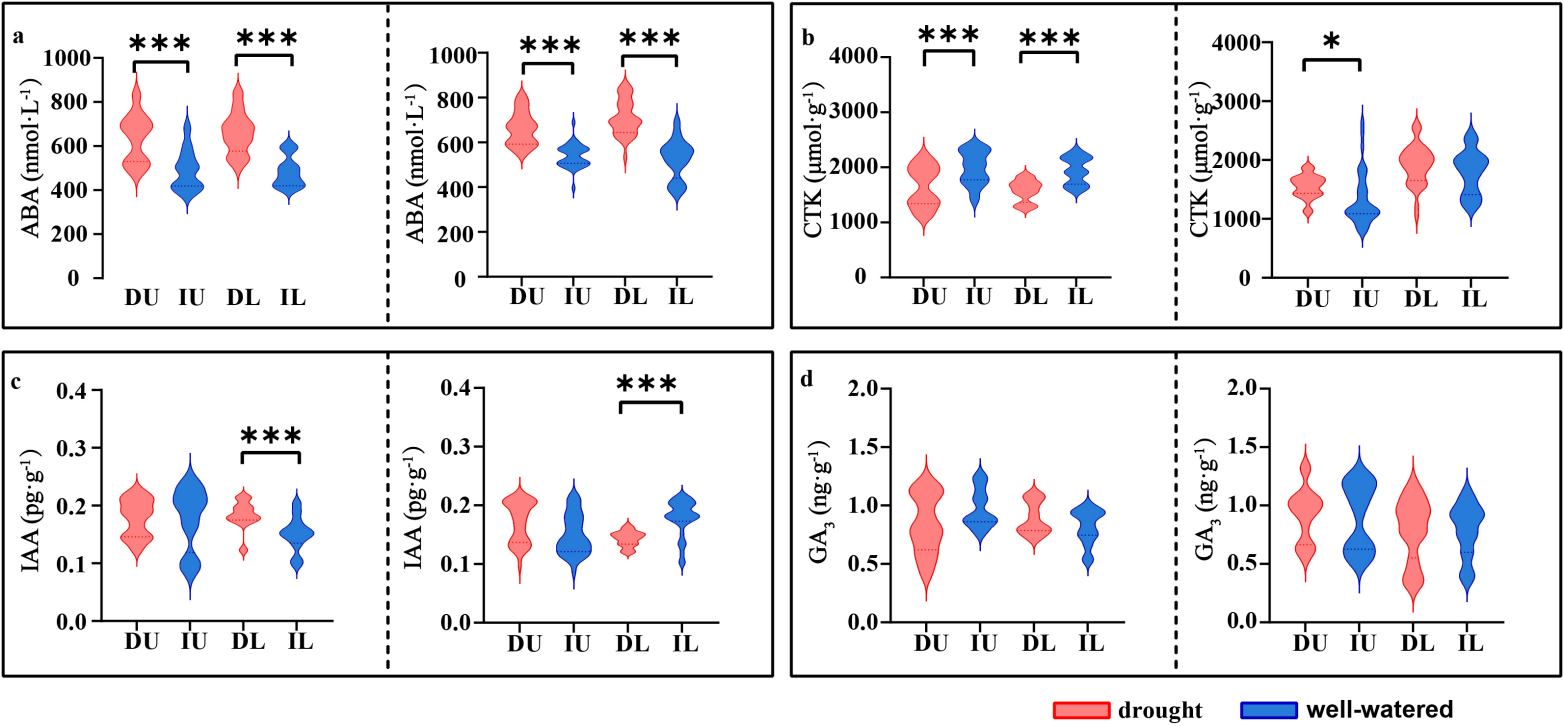
Hormone content in the upper and lower parts of the panicles of drought-tolerant and drought-sensitive cultivars under drought and irrigation treatment. **(a)** the content of ABA, with the left side representing the drought-tolerant and the right side representing the drought-sensitive (the same below); **(b)** the content of CTK; **(c)** the content of IAA; **(d)** the content of GA_3_. Red represents drought treatment, with blue represents irrigation treatment. DU represents the upper part of panicle in drought treatment, IU represents the upper part of panicle in irrigation treatment, DL represents the lower part of panicle in drought treatment, IL represents the lower part of panicle in irrigation treatment. Significance levels: *p < 0.05, ***p < 0.001.

All oat cultivars exhibited significantly higher ABA levels under drought stress compared to well-watered conditions (*p* < 0.001), consistent with the known role of ABA in drought response. Under drought stress, the CTK content significantly decreased in the drought-tolerant cultivars (*p* < 0.001), whereas a reduction occurred only in the upper panicles of the drought-sensitive cultivars (*p* < 0.05), potentially due to altered source-sink relationships. Under drought stress, the IAA content in the lower panicles increased in drought-tolerant cultivars (*p* < 0.001), while it significantly decreased in drought-sensitive cultivars (*p* < 0.001). The results indicate that IAA in drought-tolerant cultivars plays a pivotal role in modulating basal grain development under water deficit conditions (**Figure 6**).

### 3.5 The relationship between yield components and endogenous hormones

**Figure 7:**
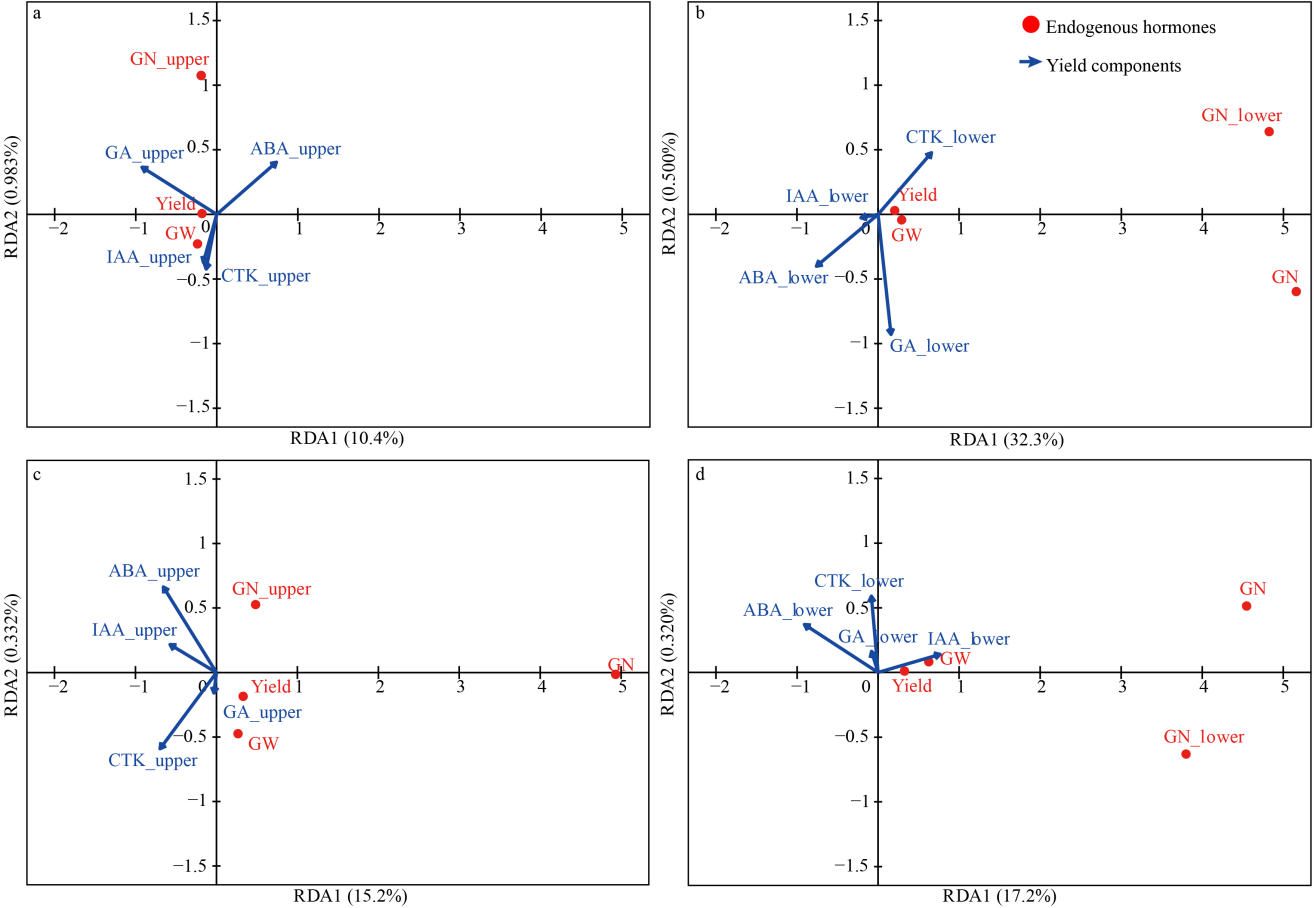
Redundancy analysis of phytohormones in different tissues of oat cultivars and their associations with grain number per panicle, grain weight, and yield. **(a)** upper panicle grain number with hormone content in drought-tolerant cultivars; **(b)** lower panicle grain number with hormone content in drought-tolerant cultivars; **(c)** upper panicle grain number with hormone content in drought-sensitive cultivars; **(d)** lower panicle grain number with hormone content in drought-sensitive cultivars. The GN_upper and GN_lower denote the grain number of upper panicle and lower panicle. The ABA_upper, CTK_upper, IAA_upper, and GA_upper denote the contents of ABA, CTK, IAA, and GA_3_ in the upper panicles, respectively, and ABA_lower, CTK_lower, IAA_lower, and GA_lower represent the hormone contents in the lower panicles.

Redundancy analysis (RDA) was employed to elucidate the relationships between hormone contents and yield components in the upper and lower parts of oat panicles for both drought-tolerant and drought-sensitive cultivars (**Figure 7**). In drought-tolerant cultivars, RDA1 and RDA2 explained 10.4% and 0.983% of the variance, respectively, for the upper panicles (**Figure 7a**). Hormones measured in the upper part of panicle and yield components are visualized. GN is strongly associated with the positive axis of RDA1, while GA_3_ and IAA exhibit negative correlations with RDA1. RDA1 and RDA2 explained 32.3% and 0.500% in the lower panicles (**Figure 7b**). Hormones from the lower part of panicle and yield components are plotted. CTK shows a strong positive loading on RDA1, whereas GA_3_ displays a negative loading. Yield and GW are tightly clustered, indicating a strong correlation between them. In drought-sensitive cultivars, RDA1 explains 15.2% of the variance, and RDA2 explains 0.332%. CTK has a strong negative loading on RDA1, while ABA and IAA show positive loadings (**Figure 7c**). Hormones from the upper part of panicle and yield components are presented. GN is on the positive RDA1 axis, suggesting a distinct relationship with other variables. RDA1 explains 17.2% of the variance, and RDA2 explains 0.320%. Lower-part hormones and yield components are depicted. CTK has a strong positive loading on RDA2, and IAA shows a positive loading on RDA1. GN is associated with the positive RDA1 axis (**Figure 7d**).

**Figure 8:**
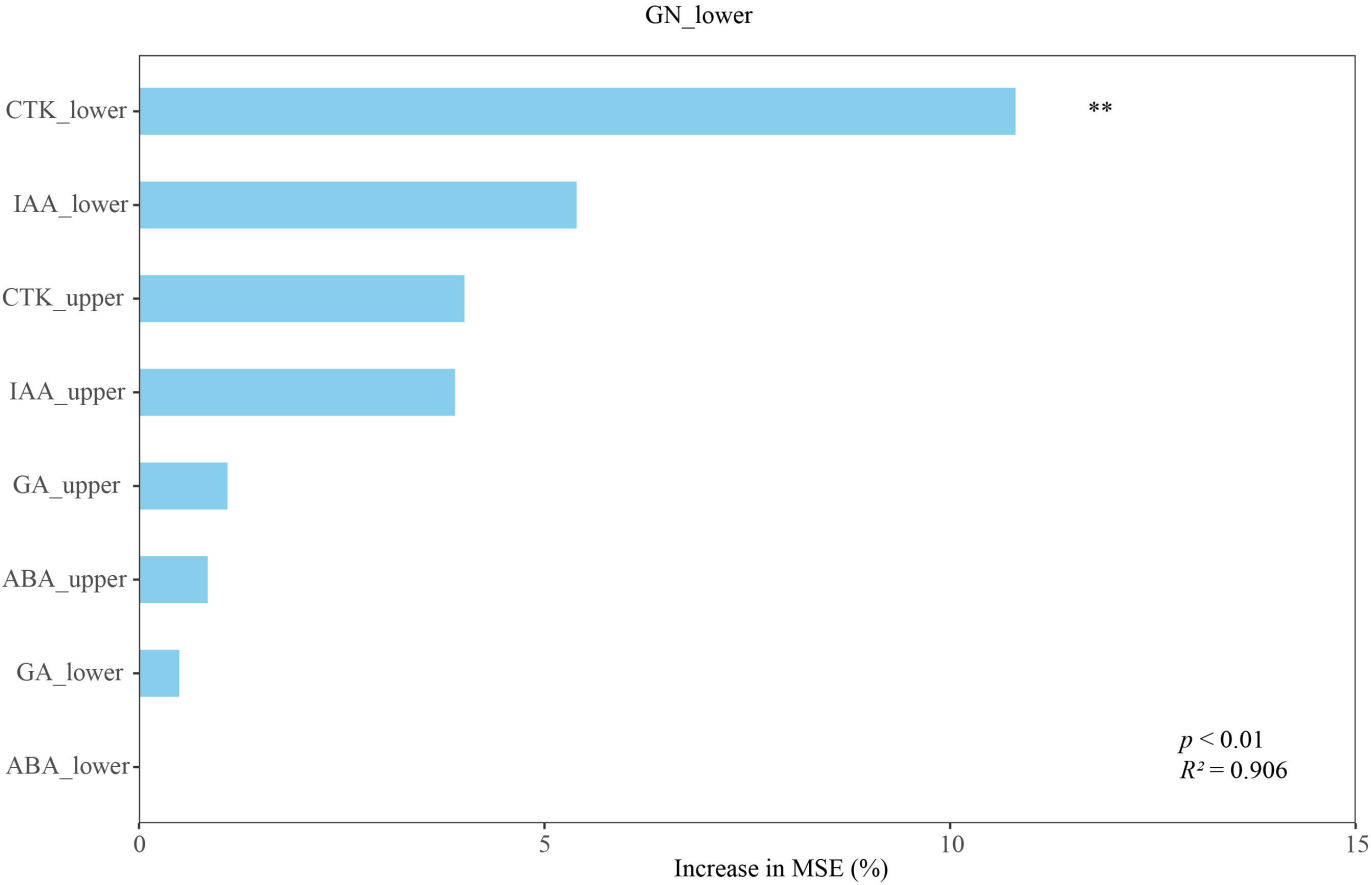
Random forest analysis of the influence of endogenous hormone contents in different panicle parts on the grain number in lower panicle. Variable importance is measured by the percentage increase in mean squared error (MSE) when a variable is permuted. The model (number of trees = 500) was constructed using hormone contents from the upper and lower panicle parts as predictors. The explanatory power of the model is indicated by the R² value, and statistical significance was determined through permutation test (*p* < 0.01). GN_lower, grain number of lower panicle, the ABA_upper, CTK_upper, IAA_upper, and GA_upper denote the contents of ABA, CTK, IAA, and GA_3_ in the upper panicles, respectively, and ABA_lower, CTK_lower, IAA_lower, and GA_lower represent the hormone contents in the lower panicles. Significance levels: **p < 0.01.

Random Forest analysis was employed to quantify the importance of phytohormones as predictive variables for the target trait. The model explained 90.63% of the variance in the data (*R²* = 0.906, *p* < 0.001). The variable importance plot, based on the percent increase in Mean Squared Error (% IncMSE), revealed substantial contributions of several hormones (**Figure 8**). The CTK (10.8%) and IAA (5.40%) contents in the lower panicles exhibited greater importance for grain number in the lower panicles than did the CTK (4.01%) and IAA (3.90%) contents in the upper panicles. CTK and IAA exerted a stronger influence on the grain number of the lower panicles compared to ABA (0-0.848%) and GA_3_ (0.496-1.09%).

## 4 Discussion

### 4.1 Relationships between yield characteristics of different panicle parts of the drought-tolerant cultivars and drought-sensitive cultivars

In this study, the most critical factor influencing oat yield was grain number per panicle rather than 1000-grain weight. This may be because, although 1000-grain weight is also an important component of yield, its influence on yield formation is more complex (**Figure 4**). Previous research has consistently identified grain number per panicle as the primary determinant of oat yield (McCabe and Burke, 2021).

Our findings demonstrate that the grain number in the lower portion of the panicle exerts a more pronounced influence on the total grain number per panicle in oats compared to that in the upper portion. This observation aligns with the physiological characteristics of oat panicle development, as the lower spikelets initiate differentiation earlier and have a longer developmental window, making them more likely to accumulate sufficient assimilates for grain formation (Liu et al., 2023). Under drought stress, drought-tolerant oat cultivars demonstrated a higher grain number on lower panicles than sensitive cultivars (**Figure 5**). This could be attributed to prioritized resource allocation in drought-tolerant cultivars. Under water limitation, oats may redirect assimilates from later-developing upper panicles to the more established lower panicles, a strategy that optimizes yield gain per unit resource. However, their superior grain number in the lower panicle region resulted in significantly higher overall yield performance. These results suggest that grain number, particularly in the lower panicle of oat, serves as a critical determinant of yield potential under water-limited conditions, which is consistent with previous findings that drought stress primarily impairs late-developing reproductive organs while having a relatively milder impact on early-initiated structures (Xie et al., 2021).

Linear regression analysis revealed that under both drought-stressed and well-watered conditions, the correlation between lower-spikelet grain number and total grains per panicle was stronger in drought-sensitive cultivars than in drought-tolerant ones (**Figure 5**). This phenomenon may be attributed to the lower grain-setting rate in the lower panicle portion of drought-sensitive cultivars due to their reduced drought tolerance, ultimately leading to a decline in total grain number per panicle. The underlying physiological mechanism could involve the impairment of vascular bundle development in drought-sensitive cultivars: studies have shown that drought stress significantly reduces the number and area of large vascular bundles in the panicle neck of sensitive cultivars, limiting the transport of photosynthates and water to the lower panicles (Xu et al., 2024).

### 4.2 Relationship between endogenous hormone content and grain number in different panicle parts

Our results revealed distinct patterns of phytohormone accumulation in oat panicles under drought stress versus irrigated conditions, which collectively shed light on the hormonal regulatory mechanisms underlying drought resistance, particularly in modulating basal grain development. The significant increase in ABA levels in all oat cultivars under drought stress (*p* < 0.01) aligns with the well-established role of ABA as a central “stress signal” in plants (**Figure 6**). Our findings also imply a potential trade-off: while ABA is essential for drought tolerance, its overaccumulation (as observed in drought-sensitive cultivars) may indirectly inhibit reproductive development, which is consistent with previous studies showing that high ABA levels during panicle development reduce pollen fertility and grain set by disrupting cell division and assimilate allocation (Canales et al., 2021). This suggests a dual role for ABA in the oat drought response: it confers stress resistance but may pose a risk to yield formation, highlighting the need for tight hormonal homeostasis in drought-tolerant cultivars.

Under drought stress, CTK content decreased across the entire panicle in drought-tolerant oat cultivars (*p* < 0.001), in contrast to sensitive cultivars, where the reduction occurred exclusively in the upper panicle (*p* < 0.05) while remaining stable in the lower panicle (**Figure 6**). This spatially divergent response in sensitive cultivars highlights a critical limitation in their adaptive strategy. The preserved CTK levels in the lower panicle, while seemingly beneficial, may actually reflect a failure to initiate systemic signaling for resource reallocation under water deficit. In contrast, the overall reduction of CTK throughout the panicle of drought-tolerant cultivars suggests a deliberate, plant-wide strategy to optimize resource use (Li et al., 2016). By downregulating this key growth-promoting hormone, tolerant cultivars potentially redirect assimilates away from less critical sinks, thereby conserving resources for the maintenance of basal oat development-a crucial determinant of final yield under drought stress (Zhang et al., 2021). This coordinated response likely contributes to their superior drought performance by establishing a more sustainable balance between growth and defense.

Most notably, drought-tolerant cultivars maintained higher IAA levels in lower panicles under drought stress (*p <* 0.001), while sensitive cultivars showed inverse patterns (*p* < 0.001) directly demonstrating that IAA-mediated regulation is a key trait distinguishing drought-tolerant from sensitive oats (**Figure 6**). IAA plays a pivotal role in panicle development by promoting cell elongation, vascular bundle formation, and the establishment of sink priority (Yu et al., 2024). For lower panicles, which initiate development earlier and rely more on stable resource supply, higher IAA levels in tolerant cultivars likely enhance sink strength through two mechanisms: (1) promoting vascular bundles development in the panicle neck, thereby improving assimilate transport to lower panicles (Deng et al., 2021); and (2) upregulating auxin-responsive genes involved in grain filling, such as those encoding sucrose transporters and starch synthases (Zhao et al., 2022). In contrast, the inverse IAA pattern in sensitive cultivars where IAA levels decrease under drought would impair these processes, leading to reduced grain set in lower panicles and ultimately lower yield.

This study uncovered a conserved hormonal regulatory module in the lower panicles that is fundamental to oat’s response to drought. Notably, irrespective of the cultivar’s overall tolerance, the grain number of the lower panicles was consistently promoted by IAA and suppressed by CTK (**Figures 7**, **8**). This consistent pattern suggests the existence of a core, position-specific regulatory mechanism for grain setting that may be inherent to oat development, even under stress conditions. The critical distinction between the drought-tolerant and sensitive cultivars lies in their dynamic hormonal adjustments. Under drought stress, the tolerant cultivar exhibited a strategic hormonal reallocation: a significant decrease in CTK content (the inhibitory signal) coupled with an increase in IAA (the promotive signal) in the lower panicles. This synergistic shift reducing suppression while enhancing promotion creates an optimal hormonal milieu that actively fosters grain set in the lower panicles, thereby contributing to yield stability. Conversely, the hormonal profile in the drought-sensitive cultivar was less favorable. The observed decrease in IAA content in the lower panicles, despite its promotive role, implies a failure to activate or maintain this crucial positive signal under stress. Consequently, while the regulatory rule (IAA-positive, CTK-negative) remains intact, the inability to accumulate beneficial IAA levels, potentially combined with a less pronounced reduction in inhibitory CTK, leads to a compromised grain-setting capacity in the lower panicles (Sharma et al., 2023). This dysregulation of hormonal homeostasis underscores a key physiological basis for its sensitivity.

### 4.3 Uncertainties and implications

While our study provides strong statistical evidence linking the IAA-CTK hormonal profile in lower panicles to drought tolerance, we acknowledge several limitations that point to future research directions. Although the identified correlations are robust and interpreted in the context of known hormonal functions, they do not establish direct causality. Future manipulative experiments, such as the exogenous application of IAA and CTK biosynthesis inhibitors to the lower panicles of contrasting cultivars under drought, are essential to definitively establish causality. Our current model is primarily physiological; the underlying genetic and molecular mechanisms governing this specific hormonal redistribution remain elusive. A comprehensive investigation, integrating transcriptomic and proteomic analyses of the lower panicle tissue, could unveil the key genes and signaling pathways involved. The practical application of these hormonal traits as biomarkers in breeding programs requires validation across a wider range of genotypes and diverse environmental conditions to assess their stability and predictive power. Addressing these points will be crucial for translating our findings into tangible strategies for oat improvement.

## 5 Conclusions

**Figure 9:**
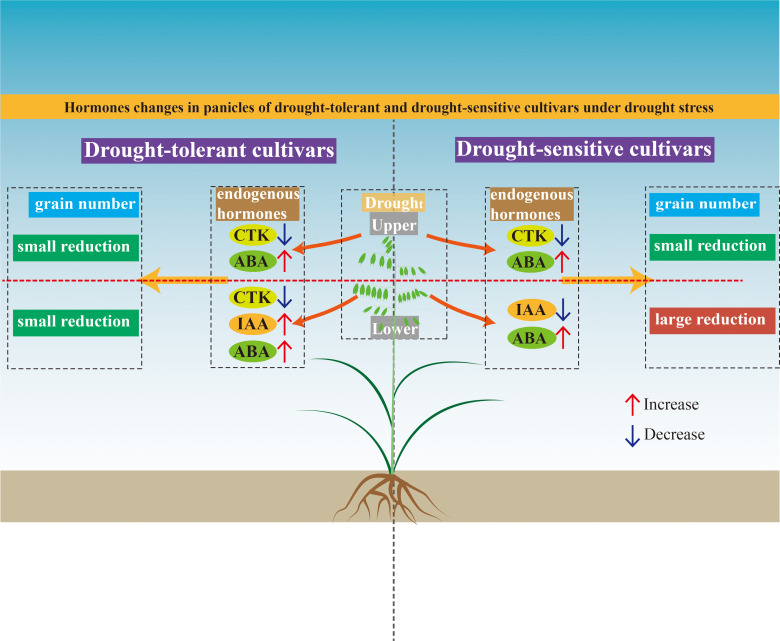
Schematic diagram summarizing the differences in endogenous hormone changes and grain number responses in panicle upper and lower spikelets of drought-tolerant and drought-sensitive oat cultivars under drought stress. Under drought conditions, drought-tolerant cultivars maintained only small reductions in grain number in both upper and lower panicle positions. This was associated with moderate increases in abscisic acid (ABA) and decreases in cytokinin (CTK) across panicle positions, as well as an increase in indole-3-acetic acid (IAA) specifically in the lower panicle. In contrast, drought-sensitive cultivars showed a large reduction in grain number in the lower panicle, accompanied by decreased CTK and increased ABA in the upper panicle, and decreased IAA and increased ABA in the lower panicle. Arrows indicate the direction of change under drought stress relative to well-watered conditions: red upward arrows = increase, blue downward arrows = decrease.

The ten oat cultivars were categorized into two distinct groups: drought-tolerant (DI ≥ 0.670) and drought-sensitive (DI ≤ 0.574). Lower panicle grain number is a key determinant of drought tolerance in oat. Drought-tolerant cultivars exhibited a smaller reduction in grain number on the lower panicles compared to the drought-sensitive cultivars. The hormonal regulation of grain number under drought stress exhibited distinct patterns. In the drought-tolerant cultivars, the grain number of the lower panicles was positively regulated by IAA but negatively regulated by CTK. Notably, the regulatory role of IAA (positive) and CTK (negative) on the grain number of the lower panicles was consistent in different cultivars. The random forest model analysis revealed that the IAA and CTK in the lower panicles exerted significantly stronger effects on grain number determination compared to their counterparts in the upper panicles. Furthermore, variations in IAA and CTK content were more predictive of grain number in the lower panicles than in the upper panicles (**Figure 9**). This study reveals a hormonal regulatory mechanism in the lower panicles of oat that is critical for drought adaptation, providing a theoretical basis for future breeding programs aimed at enhancing drought tolerance.

## Data Availability

The raw data supporting the conclusions of this article will be made available by the authors, without undue reservation.

## References

[B1] Berro I. Varela J. Gutiérrez L. (2023). An image-based methodology to evaluate oat panicle architecture. Crop Sci. 63, 648–661. doi: 10.1002/csc2.20884 41531421

[B2] Canales F. J. Rispail N. García-Tejera O. Arbona V. Pérez-de-Luque A. Prats E. (2021). Drought resistance in oat involves ABA-mediated modulation of transpiration and root hydraulic conductivity. Environ. Exp. Bot. 182, 104333. doi: 10.1016/j.envexpbot.2020.104333 38826717

[B3] Chen Y. Wang Y. Chen H. Xiang J. Zhang Y. Wang Z. . (2023). Brassinosteroids mediate endogenous phytohormone metabolism to alleviate high temperature injury at panicle initiation stage in rice. Rice Sci. 30, 70–86. doi: 10.1016/j.rsci.2022.05.005 38826717

[B4] Cho N. H. Woo O. G. Kim E. Y. Park K. Seo D. H. Yu S. G. . E3 ligase AtAIRP5/GARU regulates drought stress response by stimulating SERINE CARBOXYPEPTIDASE-LIKE1 turnover. Plant Physiol. (2022) 190, 274–289. doi: 10.1093/plphys/kiac289 PMC9434184 35699505

[B5] Deng Y. Yu Y. Hu Y. Ma L. Lin Y. Wu Y. . (2021). Auxin-mediated regulation of dorsal vascular cell development may be responsible for sucrose phloem unloading in large panicle rice. Front. Plant Sci. 12, 630997. doi: 10.3389/fpls.2021.630997 33719303 PMC7947352

[B6] Fang J. Zhan Y. Zhao B. Zhao Y. Chen Y. Zhou Q. . (2024). Photosynthetic performance and carbon metabolism in the ear organs of oats under drought stress. Front. Plant Sci. 15, 1463284. doi: 10.3389/fpls.2024.1463284 39906225 PMC11790581

[B7] Fei N. Liu J. Mi J. Wang X. Li X. Zhao B. . (2025). Transcriptome and co-expression network analyses reveal the mechanisms of early response to drought stress in oat leaves. Food Qual. Saf. 9, 16. doi: 10.1093/fqsafe/fyaf016 40388063

[B8] Steiner F. Zuffo A. M. Zoz T. Zoz T. Zoz A. Zoz J. . (2017). Drought tolerance of wheat and black oat crops at early stages of seedling growth. Rev. Ciências Agrárias 40, 576–586. doi: 10.19084/RCA16118

[B9] Girija A. Canales F. J. Haddadi B. S. Dye R. Corke F. Williams K. . (2025). Metabolomic approaches suggest two mechanisms of drought response post-anthesis in Mediterranean oat (Avena sativa L.) cultivars. Physiol. Plant 177, e70181. doi: 10.1111/ppl.70181 40148256 PMC11949858

[B10] Haverroth E. J. Musa F. A. Coelho F. K. Duarte V. D. Pereira M. B. Pacheco M. T. . (2021). Dissection of grain weight across the oat panicle. Agron. J. 113, 1492–1502. doi: 10.1002/agj2.20550 41531421

[B11] Hsu P. Dubeaux G. Takahashi Y. Schroeder J. I. (2021). Signaling mechanisms in abscisic acid-mediated stomatal closure. Plant J. 105, 307–321. doi: 10.1111/tpj.15067 33145840 PMC7902384

[B12] Hussain S. Nanda S. Zhang J. Rehmani M. I. A. Suleman M. Li G. . (2021). Auxin and cytokinin interplay during leaf morphogenesis and phyllotaxy. Plants 10, 1732. doi: 10.3390/plants10081732 34451776 PMC8400353

[B13] Li W. Herrera-Estrella L. Tran L. P. (2016). The Yin-Yang of cytokinin homeostasis and drought acclimation/adaptation. Trends Plant Sci. 21, 548–550. doi: 10.1016/j.tplants.2016.05.006 27270336

[B14] Li H. Tang Y. Meng F. Zhou W. Liang W. Yang J. . (2025). Transcriptome and metabolite reveal the inhibition induced by combined heat and drought stress on the viability of silk and pollen in summer maize. Ind. Crops Prod. 226, 120720. doi: 10.1016/j.indcrop.2025.120720 38826717

[B15] Liang X. Zhou Y. Xu W. Liang J. (2025). An intracellular CPK-ECA1 phosphoregulatory circuit couples calcium signatures to ABA homeostasis for plant osmosensivity. Sci. Adv. 11, eadz2428. doi: 10.1126/sciadv.adz2428 41032601 PMC12487894

[B16] Liu N. Li W. Qin Y. Yun Y. Yan J. Sun Q. . (2025). Comprehensive co-expression network reveals the fine-tuning of AsHSFA2c in balancing drought tolerance and growth in oat. Commun. Biol. 8, 393. doi: 10.1038/s42003-025-07857-8 40057657 PMC11890764

[B17] Liu W. Zhao L. S. Chen Y. K. Shen Y. F. Luo Z. J. Chen Y. B. . (2023). Soil properties and silage quality in response to oat and pea seeding ratios and harvest stage on the Qinghai-Tibetan Plateau. Front. Sustain. Food Syst. 7, 1143431. doi: 10.3389/fsufs.2023.1143431

[B18] Ma X. Wang W. Zhang J. Jiang Z. Xu C. Zhu W. . (2025). NRT1.1B acts as an abscisic acid receptor in integrating compound environmental cues for plants. Cell 188, 5231–5248.e20. doi: 10.1016/j.cell.2025.07.027 40795855

[B19] McCabe C. P. Burke J. I. (2021). Oat (Avena sativa) yield and grain fill responses to varying agronomic and weather factors. J. Agric. Sci. 159, 90–105. doi: 10.1017/S0021859621000320 41292463

[B20] Nan J. Ling Y. An J. Wang T. Chai M. Fu J. . (2023). Genome resequencing reveals independent domestication and breeding improvement of naked oat. GigaScience 12, 61. doi: 10.1093/gigascience/giad061 37524540 PMC10390318

[B21] Neupane D. Osborne S. Schneider S. K. Ewing P. M. (2025). Drought severity and duration effects oat yield and yield components. Agron. J. 117, e70225. doi: 10.1002/agj2.70225 41531421

[B22] Ohlert T. Smith M. D. Collins S. L. Knapp A. K. Dukes J. S. Sala O. . (2025). Drought intensity and duration interact to magnify losses in primary productivity. Science 390, 284–289. doi: 10.1126/science.ads8144 41100624

[B23] Peleg Z. Reguera M. Tumimbang E. Walia H. Blumwald E. (2011). Cytokinin-mediated source/sink modifications improve drought tolerance and increase grain yield in rice under water-stress. Plant Biotechnol. J. 9, 747–758. doi: 10.1111/j.1467-7652.2010.00584.x 21284800

[B24] Rashid A. Achary V. M. M. Abdin M. Z. Karippadakam S. Parmar H. Panditi V. . (2024). Cytokinin oxidase2 deficient mutants improves panicle and grain architecture through cytokinin accumulation and enhance drought tolerance in indica rice. Plant Cell Rep. doi: 10.1007/s00299-024-03289-6 39096362

[B25] Shanmugaraj N. Rajaraman J. Kale S. Kamal R. Huang Y. Thirulogachandar V. . (2023). Multilayered regulation of developmentally programmed pre-anthesis tip degeneration of the barley inflorescence. Plant Cell 35, 3973–4001. doi: 10.1093/plcell/koad164 37282730 PMC10615218

[B26] Sharma A. Gupta A. Ramakrishnan M. Ha C. V. Zheng B. Bhardwaj M. . (2023). Roles of abscisic acid and auxin in plants during drought: A molecular point of view. Plant Physiol. Biochem. 204, 108129. doi: 10.1016/j.plaphy.2023.108129 37897894

[B27] Steiner F. Zuffo A. M. Zoz T. Zoz A. Zoz J. (2017). Drought tolerance of wheat and black oat crops at early stages of seedling growth. Rev. Cienc. Agrar. 40, e16118. doi: 10.19084/RCA16118

[B28] Waadt R. Seller C. A. Hsu P. Takahashi Y. Munemasa S. Schroeder J. I. (2022). Plant hormone regulation of abiotic stress responses. Nat. Rev. Mol. Cell Biol. 23, 680–694. doi: 10.1038/s41580-022-00479-6 35513717 PMC9592120

[B29] Warzecha T. Bocianowski J. Warchoł M. Bathelt R. Sutkowska A. Skrzypek E. (2023). Effect of soil drought stress on selected biochemical parameters and yield of oat × maize addition (OMA) lines. Int. J. Mol. Sci. 24, 13905. doi: 10.3390/ijms241813905 37762208 PMC10531036

[B30] Xie H. Li M. Chen Y. Zhou Q. Liu W. Liang G. . (2021). Important physiological changes due to drought stress on oat. Front. Ecol. Evol. 9, 644726. doi: 10.3389/fevo.2021.644726

[B31] Xu Y. Jiang L. Gao J. Zhang W. Zhang M. Liu C. . (2024). Molecular regulation of photosynthetic carbon assimilation in oat leaves under drought stress. Plants 13, 3317. doi: 10.3390/plants13233317 39683110 PMC11644273

[B32] Yang J. Zhang J. Wang Z. Zhu Q. Wang W. (2001). Hormonal changes in the grains of rice subjected to water stress during grain filling. Plant Physiol. 127, 315–323. doi: 10.1104/pp.127.1.315 11553759 PMC117987

[B33] Yu Y. Xu X. Hu Y. Ding Y. Chen L. (2024). Indole-3-acetic acid (IAA) and sugar mediate endosperm development in rice (Oryza sativa L.). Rice 17, 66. doi: 10.1186/s12284-024-00745-5 39443408 PMC11499519

[B34] Zhang D. Cheng Y. Lu Z. Wang J. Ye X. Zhang X. . (2021). Global insights to drought stress perturbed genes in oat (Avena sativa L.) seedlings using RNA sequencing. Plant Signal. Behav. 16, 2, 1845934. doi: 10.1080/15592324.2020.1845934 33356830 PMC7849742

[B35] Zhang X. Meng W. Liu D. Pan D. Yang Y. Chen Z. . (2024c). Enhancing rice panicle branching and grain yield through tissue-specific brassinosteroid inhibition. Science 383, eadk8838. doi: 10.1126/science.adk8838 38452087

[B36] Zhang L. Zhao H. Wan N. Bai G. Kirkham M. B. Nielsen-Gammon J. W. . (2024b). An unprecedented fall drought drives Dust Bowl-like losses associated with La Niña events in US wheat production. Sci. Adv. 10, eado6864. doi: 10.1126/sciadv.ado6864 39083607 PMC11290491

[B37] Zhao Z. Wang C. Yu X. Tian Y. Wang W. Zhang Y. . (2022). Auxin regulates source-sink carbohydrate partitioning and reproductive organ development in rice. Proc. Natl. Acad. Sci. U.S.A. 119, e2121671119. doi: 10.1073/pnas.2121671119 36037381 PMC9457257

